# Immune Dysregulation in Autism Spectrum Disorder: What Do We Know about It?

**DOI:** 10.3390/ijms23063033

**Published:** 2022-03-11

**Authors:** Maria de los Angeles Robinson-Agramonte, Elena Noris García, Jarasca Fraga Guerra, Yamilé Vega Hurtado, Nicola Antonucci, Neomar Semprún-Hernández, Stephen Schultz, Dario Siniscalco

**Affiliations:** 1 Neuroimmunology Department, International Center for Neurological Restoration, Havana 11300, Cuba; 2 Immunology Department, National Institute of Nephrology, Havana 10600, Cuba; anoris@infomed.sld.cu; 3 Microbiology Department, National Institute of Nephrology, Havana 10600, Cuba; jarasca@infomed.sld.cu; 4 Research Center, International Center for Neurological Restoration, Havana 11300, Cuba; yvega@neuro.ciren.cu; 5 Biomedical Centre for Autism Research and Treatment, 70126 Bari, Italy; info@antonucci.eu; 6 Research Division, Autism Immunology Unit of Maracaibo, Catedra Libre de Autismo, Universidad del Zulia, Maracaibo 4001, Venezuela; neomar.semprun@gmail.com; 7 Department of Cellular and Integrative Physiology, Center for Biomedical Neuroscience, University of Texas Health San Antonio, San Antonio, TX 78229, USA; stevendri0629@gmail.com; 8 Department of Experimental Medicine, University of Campania, 80138 Naples, Italy; dario.siniscalco@unicampania.it; 9 European Biomedical Research Institute of Salerno (EBRIS), 84125 Salerno, Italy

**Keywords:** autism spectrum disorder (ASD), autoimmunity, neuropsychiatric disorders, cytokines, adaptive immunity, innate immunity, neuroimmunology, major histocompatibility complex (MHC), human leukocyte antigens (HLA) alleles, T helper (Th) cells, obesity, neurodegenerative diseases

## Abstract

Autism spectrum disorder (ASD) is a group of complex multifactorial neurodevelopmental disorders characterized by a wide and variable set of neuropsychiatric symptoms, including deficits in social communication, narrow and restricted interests, and repetitive behavior. The immune hypothesis is considered to be a major factor contributing to autism pathogenesis, as well as a way to explain the differences of the clinical phenotypes and comorbidities influencing disease course and severity. Evidence highlights a link between immune dysfunction and behavioral traits in autism from several types of evidence found in both cerebrospinal fluid and peripheral blood and their utility to identify autistic subgroups with specific immunophenotypes; underlying behavioral symptoms are also shown. This review summarizes current insights into immune dysfunction in ASD, with particular reference to the impact of immunological factors related to the maternal influence of autism development; comorbidities influencing autism disease course and severity; and others factors with particular relevance, including obesity. Finally, we described main elements of similarities between immunopathology overlapping neurodevelopmental and neurodegenerative disorders, taking as examples autism and Parkinson Disease, respectively.

## 1. Introduction

The immune system consists of a set of molecules and cells that are organized in tissues and organs while functioning close interacting to generate a protective response against invaders. Two components of immunity are recognized: innate and adaptive. The former recognizes pathogen-associated molecular patterns [1] and activates unspecialized cells to produce pro-inflammatory and regulatory cytokines, which function to mediate effector adaptive mechanisms via effector cells of adaptive immunity (Th1, Th2 Treg, and B cells) [2,3]. The role of adaptive immunity in neurodevelopmental disorders is supported by alterations in T- and B-cell subsets and (auto)antibody levels in the blood, cerebrospinal fluid (CSF), and brain tissues, during disease [2,3]. These cells cross the blood–brain barrier and secrete cytokines dependent upon the type of target antigen recognized in central nervous system (CNS) by antigen-presenting cells (APCs). T cell subtypes secrete variable cytokines that counter-regulate each other [4], and an imbalance between pro- and anti-inflammatory pathways is seen that plays an important role in the pathogenesis of neuropsychiatric disorders such as autism [3,5,6].

Autism spectrum disorder (ASD), a group of complex multifactorial neurodevelopmental disorders occurring in the first 3 years of life, is characterized by a wide and variable set of neuropsychiatric symptoms, including deficits in social communication, narrow and restricted interests, and repetitive behavior [7,8,9]. That the adaptive immune response plays a major role in autism development is demonstrated in various early reports from authors in this field [10,11,12]. As early as 1982, is was known that the immune system impacts cerebral function in autism, so immune dysregulation in this pathology is not a recent theory [10], as has been shown by several metabolic and immune mechanisms impacting cerebral function and the behavioral impairments core in neuropsychiatry disorders [11,12]. This review summarizes current state-of-the-art insights into immune dysfunction in ASD, with particular reference to the impact of related immunological factors, including maternal influence regarding the risk of autism development as well as comorbidities influencing the autism disease course. This paper also presents several characteristics of immune-dysregulation-associated maternal obesity and the link between obesity, inflammation, and neurobehavioral outcomes.

## 2. Methods

**Figure 1:**
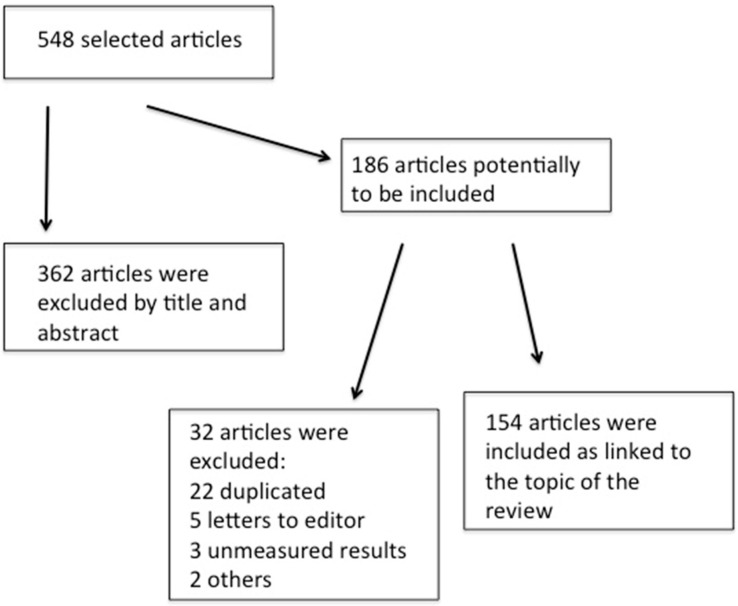
Included and excluded references screened for the literature review.

The data in this review were obtained from a comprehensive search of PubMed under the followings terms as key words: autism, autism spectrum disorders, autistic children associated with autoimmunity, inflammation, neuroinflammation, immunology, immunological dysfunction, immunomodulation, neuroimmunology, cytokines, antibody, autoantibodies, immunoglobulins, major histocompatibility complex (MHC), lymphocyte, glial activation, microglia, neurodegenerative disorders, obesity, and maternal obesity. English and Spanish articles were included. Figure 1 displays the number of the included and excluded references screened for the literature review.

## 3. Genetic Factors and Immunological Disturbance in Autism Spectrum Disorders

### 3.1. Major Histocompatibility Complex (MHC) and Autism Spectrum Disorder

The MHC is a highly polymorphic cluster of genes with some of the greatest allelic diversity in the genome. MHC genes are both polygenic (containing multiple genes) and polymorphic (containing multiple variants of each gene). It is well known that MHC proteins mediate both the adaptive and innate immune responses [13,14,15]. There are multiple studies supporting the association of different genes with ASD development, which also involve the function of the immune system. The human leukocyte antigen (HLA) alleles A2, Death Receptor (DR)4, and DR11 are associated with a diminished lymphocyte response and are involved in a major susceptibility for ASD [16,17,18,19,20]. Within the HLA class III region, there is a complement C4B null allele, resulting from duplications of C4A, that confers a relative risk of 4.3 for the development of ASD [21,22]. In addition, the serine and threonine kinase C gene PRKCB1, which is involved in both B-cell activation and neuronal function, has been linked to ASD in some studies [23,24,25,26]. Peripheral blood RNA expression studies demonstrated an upregulation of genes involved in innate immune activation through the natural killer (NK) pathway in individuals with ASD, a finding that was confirmed in functional studies using NK-cells [27]. Multiple altered gene-susceptibility, related to innate immune activation and the loss of adaptive immune regulation, has been suggested. For example, a disrupted transcription of phosphatase and tensin homolog (PTEN) genes (involved in T regulatory cell development) and reelin, the association of a met genetic variant with autism-associated maternal autoantibodies reactive to fetal brain proteins, and cytokine expression have been associated with ASD etiology [28,29].

On the other hand, the function of brain MHC molecules’ expression and their role in CNS development and plasticity is less understood [30]. Although MHC class I (MHC-I) protein was historically thought to be absent from the surface of neurons, more current evidence indicates that MHC-I protein is expressed on the surface of axons and dendrites under a regulated distribution [19,31]. For example, MHC-I protein is located at synapses, both pre- and post-synaptically [29] and controls axonal and dendritic outgrowth. It also regulates, in a negative way, the initial establishment of connections in the CNS and regulates, in multiple ways, synaptic transmission in hippocampal and cortical neurons. The balance of excitation to inhibition on cortical neurons is altered by changing MHC-I levels [32], which from all these studies shows that MHC-I proteins bidirectionally regulate both the initial establishment and strength of synapses in the CNS. It accomplishes this in a region-specific manner and mediates homeostatic plasticity [33,34].

The role of MHC-I in limiting neural connectivity and function also may have deep implications for neurodevelopmental disorders and neuropsychiatric diseases [35]. Specific MHC-I haplotypes and mutations in MHC-I genes have been implicated in ASD [21,22]. MHC-I levels on neurons are regulated by cytokines [34,36,37,38], and cytokine levels are altered in the blood, brain, and cerebrospinal fluid (CSF) in many neurodevelopmental disorders [39,40,41,42]. This suggests a peripheral immune response influencing brain cytokines via blood–brain barrier transference in early development, which becomes an altered connectivity and/or function in the CNS through changes in MHC-I levels in disease. Functional polymorphisms of macrophage inhibitory factor (MIF), which has several effects on innate and adaptive immune responses, have also been associated with ASD individuals [41,43]. Increased sera concentrations of MIF are correlated with worsening behavioral assessments in individuals with ASD compared with their unaffected siblings [44]. Moreover, genes that can affect immune responses, such as PTEN and reelin, have been associated with ASD [45,46,47,48,49], suggesting that multiple susceptibility genes related to innate immune activation and/or the loss of adaptive immune regulation may be involved in the etiology of ASD.

### 3.2. Adaptive Immune Reaction in Autism Spectrum Disorders

#### 3.2.1. Cellular Immune Reaction in Autism

Earlier observations of immune pathology in ASD described a decreased lymphocyte response to mitogens in children with ASD and demonstrated the involvement of lymphocyte subsets in ASD pathology, finding a decreased number of these populations and an imbalanced ratio of helper/suppressor cells in these individuals [18,26]. Other studies confirming a lower helper/suppressor ratio with a decreased percentage of helper–inducer T cells and decreased percentage of cells positive to interleukin (IL)-2R under mitogenic stimulation were carried out to show an inversely correlated relationship with severity of autistic traits [7,50]. A significant increase in CD4+ memory and decrease in CD4+ naïve T cells associated with HLA A2-DR11 have also been observed in autism [51], as well as an imbalance of cytokines produced by CD4+ and CD8+ T cells with reported skewing toward Th2 response. This underlies a reduced proportion of CD4+ and CD8+ T cells producing IFN-γ and IL-2, which is in contrast with those T cells releasing other suppressor cytokines such as IL-4 [50,52]. T regulator cells (Tregs) play a key role in regulation of immune responses. Studies revealing a lower number of CD4+ CD25 high Tregs in the blood of autistic children were reported by Mostafa and colleagues. This supports the reporting of allergic problems and family history of autoimmunity as risk factors for ASD since lower numbers of CD4+ CD25 high Tregs are seen in individuals with autism [51,52,53]. Dysregulation of Th1, Th2, Th17, and Treg-related transcription factors, and a deficit of forkhead box protein 3+ Tregs in combination with up-regulation of Th1/Th2/Th17-related transcription factors, were also described [41,52,53]. 

In the context of T- lymphocyte dysregulation occurring in a high number of autism subjects, the imbalances involved the abnormal T helper-suppressor cells ratio [54,55,56,57], a systemic deficit of regulatory T cells [36], and dysregulated cytokine release in ASD [58]. Therefore, the Th-17 lymphocytes subpopulation becomes relevant in the immune pahology this disorder.

In the interface between peripheral and CNS immunity, specific lymphocyte-derived cytokines impacting brain development, neural function, and behavior from specific subsets of lymphocytes work together to generate heterogeneous immune responses in brain [59]. Brain–peripheral interactions in neurodevelopmental disorders impact tissues via effector cytokines, such as IL-17A and IL-22. IL-17 is a critical mediator of neurodevelopmental abnormalities associated with maternal immune activation (MIA), and it has been found that IL-17 produced by maternal Th17 cells induces cortical malformations [60]. In addition, social behavioral defects mediated by the maternal microbiome and the gut microbiome are an increasingly recognized modulators of peripheral immune responses, and by extension of the brain, which promotes Th17 cell differentiation [38,56,61,62]. Similarly, the proinflammatory IL-6 cytokine derived from the placenta also has an impact on social behavior in autism [63,64].

In addition, Th17 CD4+ T cells are thought to be important players in autoimmune and neuroinflammatory diseases, and their product, IL-17A, is known to be up-regulated in several autoimmune systemic and neurological diseases [65,66,67]. Additionally, studies from different groups reveal significant changes to IL-17A in ASD with a positive correlation to severity [55], and the highest percentage of children are shown to share a severe form of the disease [68]. Upregulation of IL-17 was also found in ASD children with concomitant asthma [66,67], while the IL-23 cytokine, known to induce Th17 cell secretion of IL-17, was inversely found to be down-regulated in children at ASD onset, although it was positively associated with more impaired behavioral scores [54,69].

Frequently, ASD individuals are reported to have a higher concentration of proinflammatory or lower concentration of anti-inflammatory cytokines [55,70,71]. Mainly, the results show the pro-inflammatory cytokine profile in ASD, which involves IL-1 upregulation in connection with regression and is associated with enhanced level of IL-5 and IL-17 [72,73], which are involved in autoimmunity. Other changes in cytokine levels in ASD have been reported to be associated with severity, deficits in social sphere, impaired adaptive skills, and development [55]. Other studies have shown associations with hyperactivity, lethargy, and irritability [7,74,75,76]. IL-6 was strongly associated with ASD severity and deficits in social sphere, as well as a much greater alteration of intellectual quotient in those children with lower maximum levels [7,55,77]. Additionally, IL-6 is up-regulated, similar to IL-1, and has a significant correlation with hyperactivity, lethargy, and irritability [78].

Although without general consensus, ASD individuals are thought to have a higher concentration of pro-inflammatory or lower concentration of anti-inflammatory cytokines than healthy controls, as well as changes in S100 beta protein linked to glial function from peripheral blood analysis [55,79,80,81]. Some of these studies analyzed the correlation of behavioral profiles with immune abnormalities, ASD severity, and cytokine or chemokine abnormalities: an increased concentration of IL-1β, IL-6, IL-12p70, and TNF-α has been found [7,55,71,81]. In this context, IL-6 correlated positively with social impairments [82].

On the other hand, up-regulation of IL-1β, IL-10, monocyte chemoattractant protein (MCP), IL- 23, transforming growth factor (TGF)-β1, tumor necrosis factor (TNF)-α, and granulocyte macrophage colony-stimulating factor (GM-CSF)-1 were also described in relation to social dysfunction from several research groups [7,83,84,85]. Furthermore, stereotypic behavior, a core symptom of autism, seemed to correlate with down-regulation of TGF-β1 and GM-CSF, as well as up-regulation of IL-1β, IL-6, IL-8, IL12p40, TNF-α, and interferon (IFN)-γ [83,84]. Surprisingly, exacerbations of hyperactivity were found to be linked to low levels of anti-inflammatory cytokines (IL-10 and TGF-β), high levels of pro-inflammatory cytokines (IL-1β and IL-6) [71,79], several chemokines (IL-8, RANTES, and eotaxin), low levels of chemokine ligand (CXCL)-5 and IL-13, and high levels of IL-12p40 [83,86]. An interesting observation about sleep disturbances and aggressive behavior in ASD was made by Careaga et al., who found up-regulation of IL-1β, IL-6, IL-10, and MCP-1 in these ASD children, and, in particular, Th-1 skewed response was associated with more severe developmental impairment [7].

Understanding the differences and interactions between the peripheral nervous system and CNS is crucial for determining novel therapeutic strategies in ASD. A relevant insight into ASD pathogenesis was achieved through cytokine studies on autistic brains, as well as several immune phenotypes derived from the studies of peripheral blood soluble factors that correlate with increased or/and severe behavioral impairments of the disorder [10]. In this context, it has been concluded that ASD may be linked to a disturbed immune balance involving both of the main dysregulations focused on pro-inflammatory mediators, as well as anti-inflammatory cytokines and autoimmunity [11].

#### 3.2.2. Adaptive Immune Response in Autism

##### Antibody Reaction in ASD Brain Tissue

In addition, abnormal immune responses in brain tissue have been demonstrated by an altered reaction to antigens, such as human myelin basic protein (MBP), in autistic brain, and the detection of enhanced MBP and anti-serotonin 5HT1A receptor autoantibodies in brain tissue. Although without a total consensus from different research groups, it has been suggested that there is a connection between autism, serotonin, and MBP [82,86,87]. Other laboratory data support the autoimmune hypothesis in the pathogenesis of autism based on the cerebrospinal fluid findings of elevated levels of autoantibodies to MBP and serological antibodies to measles virus, supporting the autoimmune pathogenesis of the disorder [88,89]. The detection of significantly increased autoantibodies to glial fibrillary acidic protein and to neuro-axon filament protein in autistic patients, as well as a particular association between the elevated levels of serum measles antibodies and human herpesvirus-6, with brain autoantibodies in autism is also supportive of the viral hypothesis of autoimmune response induction of ASD [90], although it is clear that the subsequent activation of cytokines is the damaging factor associated with autism [11].

##### Peripheral Immunoglobulins Response in Autism

The majority of studies assessing B-cell number and function in autism have seen abnormalities; however, some peculiarities have been published that show only a significantly higher number of B cells but not of B cell function in children with ASD [91,92]. 

A selective deficiency of serum immunoglobulin (Ig) A in both children and adults with ASD compared to controls is accompanied by an upregulated expression of CD23+ B lymphocytes in children with the regressive form of disease [76]. Plasma levels of Igs in ASD reveal a reduced level of IgG and IgM isotypes inversely correlated with scores on lowest IgG levels but without B-cell dysfunction [53,93]. Higher concentrations of IgA, IgG, and IgE food-specific antibodies are also described in ASD [44,94]. 

Levels of IgG4 and IgG1 subclasses are increased in ASD individuals, with differences in IgA, IgG2, and IgG3 observed between ASD children and healthy family members [62]. From these findings, the production of Ig isotype and subclasses in autism could be associated with a cytokine-related influence on autoimmune B cells but not linked to B cell dysfunction [95]. In addition, a higher frequency of D8/17 B lymphocytes was found in ASD subjects, particularly in subjects with a pattern of repetitive behaviors. It was also found that B cells were hypersensitive to the vaccine preservative thimerosal, which could be revealing in the context of vaccination in these patients [96]. In the context of autoantibodies, this impact in the brain of individuals with autism was seen in patients with more severe cognitive and behavioral profiles [94,97]. 

##### Maternal Autoantibodies Influencing Gestational Environment in Risk for ASD

The long list of serum autoantibodies in autism includes antibodies to ganglioside M1, the most abundant sialylated glycosphingolipid component of neuronal membranes. Mostafa and Al-Ayadhi (2011) found significantly higher amounts of these autoantibodies in children with ASD compared to controls, with the highest levels seen in the most severe cases of ASD [95]. These autoantibodies to gangliosides are frequently observed in autoimmune disorders associated with neurological impairment, such as SLE and Guillain-Barré syndrome [98,99]. Immunoreactivity to autoantibodies in children with ASD also include those specific to cardiolipin, phosphoserine, and α2-glycoprotein 1, as identified by Careaga and colleagues, 2013 [100], and to myelin-associated glycoprotein [62], mitochondrial DNA, double stranded DNA, nucleus, and nucleosomes [101,102,103]. 

More recently, autoantibodies against folate receptor (FRA) have been recognized as impacting brain pathology and behavioral phenotypes in children with ASD. In 2013, Frye’s research group found FRAs to be prevalent in up to 70% of children with ASD, including blocking and binding FRAs [104]. This study found improvement in communication, language, attention, and stereotypic behaviors in treated, compared with non-treated, ASD individuals [104]. This was also reported by Ramaekers et al., this year, who identified a significantly higher prevalence of blocking FRA in ASD compared to non-autistic individuals with developmental delays [105]. At the same time, it has been suggested that the immunoreactivity to autoantibodies and a relationship with familial autoimmunity impact brain pathology and behavioral phenotypes in ASD [99,106]. However, autoantibodies in autism are not only restricted to maternal phenomenology [107]. It is now believed that the maternal immune system (MIS) during fetal development is highly active and dynamic. It interacts with fetal immune cells to create a prenatal environment supporting pregnancy [108] but also influences programming of the fetal immune system [109,110]. 

During gestation, IgG antibodies are supplied to the fetus by the mother in a highly controlled manner mediated by neonatal Fc receptors (FcRn), an MHC class I (MHC-I)-related receptor [111,112]. However, during the antibodies’ transplacental exchange, the prenatal environment could become altered, affecting fetal development and changing its susceptibility to neurodevelopmental disorders such as ASD [113]. This idea has been circulating since an early report focused on the family history of autoimmune disease indicated this as a strong risk factor for autism development [48,55], particularly due to maternal autoantibodies being transferred from mother to the fetal central nervous system (CNS), which could affect fetal brain development [5,6]. 

Several studies have documented a significant relationship between autoimmune disorders and increased risk of ASD, underlying a differential risk linked to the different maternal autoimmune diseases diagnosed during pregnancy. Thus, it recently has been inferred that folate receptor autoantibodies, which are prevalent in pregnancy and in developmental disorders such as autism, can block folate transport to the fetus and ultimately to the brain in young children, contributing to the core symptoms of autism [106]. This could contribute to autism by the fetus’ exposure to maternal autoantibodies or due to an association with a family history of autoimmune disease by heritable factors. In both cases this would cause alterations in the maternal prenatal environment to drive risk for ASD [114,115]. Other evidence supports that trans placental passage of fetal brain-reactive antibodies can directly lead to ASD-like behavioral abnormalities in the offspring, such as hyperactivity and deficits in social interaction [55,116].

Further, families with at least one child with autism tend to display a high autoimmune burden such as type 1 diabetes, thyroiditis, and maternal rheumatoid arthritis. The risk of ASD in offspring is particularly increased when maternal autoimmunity is in an active phase during pregnancy [111], suggesting that an active inflammatory state during gestation may negatively influence the fetal neurodevelopmental trajectory.

As indicated above, there are a growing number of publications regarding shared genetic risk interacting with prenatal autoimmunity activation to support the immune hypothesis in autism. This risk is from the differential influence of the maternal and fetal immune environment from the different phenotypic clinical and evolutive courses of the disease. With this in mind, Meltzer and Van de Water, 2017, suggested that autism may result from maternal and/or host autoantibodies that selectively disrupt neural circuits regulating social behavior. In the context of maternal autoimmunity in ASD, several authors have revealed that a subset of mothers of children with ASD (10–12%) have been found to show immunoreactivity mediated by autoantibodies specific to fetal brain, inducing ASD-like pathology. This is seen in both human and in animal models [117,118,119,120,121]. During pregnancy, diseases such as rubella, measles, or toxoplasmosis can also negatively impact early neurodevelopment of the fetus. It was reported by studies of pregnancy that history of viral and bacterial infections occurring in the first or third trimester, respectively, or maternal fever during gestation, showed a higher risk for later development of ASD in offspring [121,122]. From these findings, common immunopathological components may be overlapping in at-risk populations to generate neurodevelopmental and neurodegenerative disorders from the potential impact of the immune system disruptions occurring in early and late stages of life. 

### 3.3. Innate Immunity in ASD

Children with ASD have adverse reactions to benign factors such as immunizations, common illnesses, and environmental challenges [48,62,71,72,73,74,75,76,77,78,79,80,81,82,84,85,86,87,88,89,90,91,92,93,94,95,96,97,98,99,100,101,102,103,104,105,106,107,108,109,110,111,112,113,115,116,117,118,119,120,121,122,123]. Clinical reports suggest that aberrant behavior might be improved in some febrile ASD children. 

#### 3.3.1. Natural Killer Cells

Natural killer (NK) cells constitute about 15% of circulating lymphocytes and play a pivotal role in the innate immune system [124]. They secrete a cytokine profile that includes interferon gamma (IFN-γ), tumor necrosis factor-alpha (TNF-α), and IL-10, which have a cytolytic function as mediators of cellular cytotoxicity [125], as well as surveillance of immune function through crosstalk with dendritic cells [40,126,127]. Imbalances between their activation and inhibitory states could also play a role in autoimmune diseases, and although a specific mechanism is not clear, it could be linked to a dysregulated proinflammatory immune response [26]. An earlier report found NK cells associated with autism were focused on reduced NK cell activity, higher absolute numbers of NK cells in peripheral blood, and an increased expression of NK cell receptor RNA. Further, production of perforin, granzyme B, and IFN-γ in blood samples from these individuals was increased [125,127]. IL-15, a major NK cell stimulant, was significantly up-regulated in children with ASD [42,54,125,128] and was further increased in those with GI disturbances [129]. IL-12 is also increased at significant levels in plasma of autistic patients showing EEG abnormalities and GI complaints [68,123], as well as low Intelligence Quotient (IQ) and prominent aberrant behavior [73,82,130].

#### 3.3.2. Monocytes

Monocytes are a part of an innate immune system and differentiate into macrophages. They migrate into the surrounding tissue, where they present antigens to lymphocytes that secrete proinflammatory mediators, such as IL-1β, IL-8 or TNF-α, in the course of systemic autoimmune or neurodegenerative disorders [2,40,131]. Increased levels of pro-inflammatory cytokines produced by peripheral blood mononuclear cells have been observed in individuals with ASD [26,68,128]. Monocytes in children with ASD may be positive for a surface receptor expressed on cells susceptible to apoptosis [75], and there is evidence that the differential LTR stimulations increase the concentration of several cytokines such as IL-1β, IL-6, and TNF-α. Other cytokines are decreased, such as IL-1β, IL-6, GM-CSF, and TNF-α, in ASD individuals, which may influence neuronal activity and drive autoimmunity [128,131,132,133,134]. Children with symptoms of irritability, lethargy, or hyperactivity have also been shown to have higher release of pro-inflammatory cytokines, as well as lower amounts of anti-inflammatory cytokine secretion, suggesting an active pro-inflammatory state is required to exacerbate the behavioral symptoms occurring in the autism disorder course [129,135].

#### 3.3.3. Microglia

Microglia are the professional resident cells of innate immunity in the brain and are specialized tissue macrophages involved in synaptic and neuronal development. They have been shown to play an important role in the pathogenesis of neuropsychiatric disorders [136,137]. The activation of microglia has been linked to abnormal brain connectivity in children with ASD [68,138,139,140], and the crosstalk between the peripheral immune elements and microglia, as well as abnormal white matter connectivity, has been described in ASD, indicating these cells as a potential source for intervention [138,139,140,141].

An aberrant response from immune cells in the CNS includes microglial cells. These cells are the resident phagocytes of the CNS implicated in neuronal cell death, which is mediated by the actions of inflammatory cytokines and neuropeptides [139,140]. The inflammatory cytokines IL-1, IL-6, and TNFα can directly affect the brain and alter neurodevelopment to impact behavior [142]. The microglia make contact with neurons and glia, and, like macrophages in other tissues, they are programmed to adopt a particular brain state [143,144] and perform critical local immune functions during development in health and disease, which is relevant to psychiatric diseases [145]. Further, astrocytes are also implicated in the pathogenesis of many psychiatric disorders, particularly by their contributions to synapse formation, function, and elimination. They are also essential to the immune response [143,144,146]. Astrocytes can both secrete and sense immune signals, and they can produce cytokines that affect the function of microglia and other brain cells, which is relevant during homeostasis and in response to inflammation [134]. Increased levels of pro-inflammatory cytokines are observed in many brain regions, and significant innate immune activation is also observed, particularly in microglia and astroglia of individuals with ASD [5,145].

Several clinical studies have revealed a strong link between a pro-inflammatory cytokine profile and ASD [70,82,130,147]. Elevated levels of pro-inflammatory cytokines and a pro-inflammatory phenotype of microglia have been demonstrated in autistic patients [18,145,148] and experimental models, such as maternal immune activation, suggesting that a pro-inflammatory response in the fetal environment can cause behavioral changes that last well into adulthood [5,67].

## 4. Cytokines and Chemokines in Brain and Peripheral Compartment in Autism

Inflammatory mechanisms involving cytokines in autism are underlined by different pathways: (1) by proinflammatory cytokines arising from maternal inflammation, infection, or allergy during pregnancy, which cross the placenta, to enter the fetal circulation and pass through the fetal blood–brain barrier (BBB) and impact fetal brain tissue; (2) by an excess of proinflammatory cytokines released into the fetal brain to cause aberrant neurogenesis, altering synaptogenesis and neuronal function; and (3) by altered brain development resulting in behavioral symptoms, including reduced sociability, stereotypic and repetitive behavior, hyperactivity, attention deficit, anxiety, and hypersensitivity to environmental stimuli [147].

From previously published papers, authors have demonstrated an increase of TNF-α, IL-6, granulocyte colony-stimulating factor (G-CSF), IFN-γ, and IL-8 in the brains of ASD patients, as well as an elevated proinflammatory Th1 cytokine profile. The Th1/Th2 ratio (measured as IFN-γ/IL-10 ratio) and IL-8 cytokine up-regulation have been noted in the peripheral compartment of autistic patients [3,41,42,75]. Some studies in plasma of individuals with ASD showed increased levels of IFN-γ, IL-2, and IL-12, suggesting a narrow pattern potentially linked to pathological stimulation of Th1 cells [21]. The increase in the Th1 inflammatory response may be linked to a high release of IL-6, interleukin-1 receptor antagonist (IL-1RA), IFN-γ, and TNF-α observed by authors in blood, as well as to interleukin-2 receptor (IL-2R) and IL-1RA found in serum of ASD individuals [149,150]. In this context, the relationship between peripheral Th1 and Th2 cytokines, also evaluated in these subjects, supports the imbalance of Th1/Th2 cytokines with increased IL-4+CD4+ T cells and IL-4+CD8+ T cells and decreased proportions of IFN-γ+CD4+ T cells, IL-2+CD4+ T cells, IFN-γ+CD8+, and IL-2+CD8+ T cells observed in children with autism [41,73]. The measured cytokine production against common dietary proteins in autism showed an increase of pro-inflammatory cytokine responses (IFN-γ and TNF-α) as a factor of susceptibility to predispose autistic children to GI inflammation and produce a worsening of disorder behavioral symptoms [81,151]. Elevated levels of IL-1β, IL-1RA, IL-5, IL-8, IL-12p70, IL-13, and IL-17 cytokines have been found in plasma of high-functioning autistic male children, and chemokine and cytokine profile alterations have been described, which are associated with the comorbidities of attention deficit hyperactivity disorder in autism [75,152,153,154].

### 4.1. Cytokines Affecting the Gestational Environment Increasing Risk for Autism

From the description above, a wide variety of different maternal cytokines are involved in autism pathology [75,155,156]. Different groups have suggested mechanisms to argue how maternal immune activation leads to long-term behavioral changes in ASD following the dysregulated production of soluble factors (IL-6, IL-1β, and IL-17) [157,158,159,160]. The majority of maternal cytokine research as a risk factor for ASD has been focused on their impact in the mediation of severe placental damage causing neurodevelopmental anomalies in their offspring [161]. In this context, clinical findings suggest that elevated IL-6 and IL-1β cytokines in the amniotic fluid and placental inflammation are substantial elements to propose as mediators and predictors of brain injury in premature infants [162].

**Figure 2:**
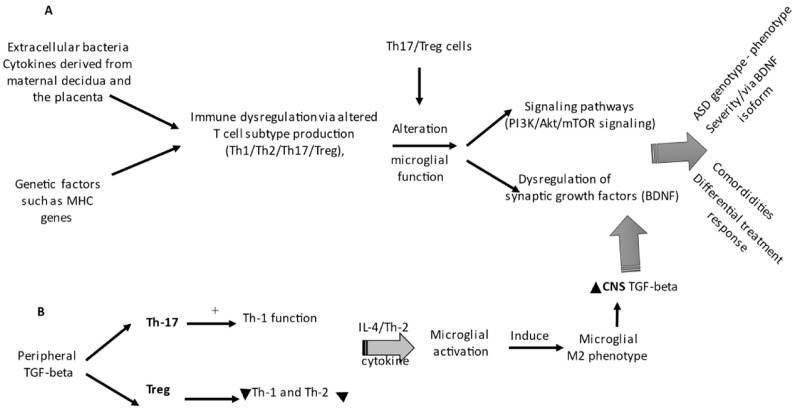
Immune dysregulation, ASD phenotypes, and treatment responses. (**A**) Soluble mediators from direct transfer across the placenta target the fetal brain and induce behavioral outcomes, differential phenotypes, and drug response in autism. (**B**) T cell subpopulations influencing microglial functioning drive synaptic growth factors, dysregulation, and signaling pathways (such as mTOR), contributing to differential clinical ASD phenotypes and treatment responses. The figure has been drawn using Power Point 2013.

An increase in IL-1β expression and other related cytokines (IL-6, TNF-α, and IL-10) are present in maternal plasma, placenta, and fetal brain [156,161,163]. The main mechanisms underlying the role of these cytokines in autism pathology include IL-1β stimulation of IL-6 production as the main effector of microglial activation after an infection or injury [164,165,166,167,168,169]. IL-6 is also an important regulator of the balance between pro-inflammatory Th17 and Treg cells at the maternal–fetal interface [115,167,170,171], Figure 2, which can occur by mediating IL-6 access to the prenatal environment by (1) direct transfer across the placenta to target the fetal brain [172]; (2) IL-6, which is not maternally derived, being produced by the fetus [173]; or (3) maternal IL-6 production following the activation of decidua immune cells, such as NK cells, macrophages, and decidual granulocytes. In addition, an elevated level of placental IL-6 may also be derived from the maternal circulation [174].

Other data suggesting mechanisms that share genetic risk derived from prenatal immune activation describe maternal infection and antepartum maternal infection becoming as important risk factors for autism linked to immune dysregulation [160]. The MIA model shows an increase in response to infectious challenges, such as immunoreactivity, in response to influenza and herpes virus. Hence, MIA could become a trigger for reducing the neonate’s IGF-1 available to aid in neo-neuronal myelination, independent of the maternal level of IGF-1. In line with this finding, a reduced placental production of IGF-1 can reduce the transfer of nutrients across the placenta to the fetus [134,175,176]. Additional evidence reported from authors confirms that the supplementation of IGF-1 in the newborn could reduce or eliminate neurologic defects, especially linked to the eventual development of ASD [177]. In addition to that, evidence for insufficient initial IGF-1 secretion assumes that the dysconnectivity characteristic of autism appears to be the result of a diminished effect of IGF-1 on neonatal neurogenesis and myelogenesis secondary to disturbances in the PI3K/AKT pathway [71,134,175,176,177].

### 4.2. Cytokines and Clinical Phenotype in ASD

Studies examining the correlation between ASD severity and cytokines or chemokines tend to confirm their importance. Consequently, an increase of IL-1β [11], IL-6, IL-12p70, TNF-α [54,79,178,179], IL-17A, macrophage-derived chemokine (MDC), thymus and activation-related chemokine (TARC) [86,118], and migration inhibitory factor (MIF) [125] were found to be correlated with the more severe behavioral symptoms, while several investigations identified a relationship between the aberrant social communication and IL-6 to be the most relevant to show a correlation with social impairments [10]. In addition, a positive correlation with social dysfunction was observed with up-regulation of IL-1β [7,36], IL-10, MCP-1 [7,180,181], MIP-1β, MIP-1δ, GM-CSF [54,182], and MIF [81,151] and down-regulation of IL-23 [83], tumor growth factor beta 1 (TGF-β1 [183,184], and TNF-α [40] in ASD. Stereotypic behavior seemed to correlate with down-regulation of TGF-β1 [184] and GM-CSF, as well as up-regulation of IL-1β, IL-6, IL-8, IL12p40, TNF-α, and IFN-γ [55,185]. A restricted pattern of behavior and interests was more pronounced in patients with a high concentration of MCP-1, RANTES, and platelet-derived growth factor subunit B (PDGF-BB) [186]. Exacerbations of both hyperactivity and lethargy were found to be linked to low levels of anti-inflammatory cytokines IL-10 and TGF-β and high levels of pro-inflammatory cytokines and chemokines IL-1β, IL-6, IL-8, RANTES, and eotaxin [75,77]. Hyperactivity was also associated with low levels of CXCL5 and IL-13 [43,86] and high levels of IL-12p40 [55]. Irritability was associated with a low level of TNF-α [82] and TGF-β1 [187]. 

A wide range of cytokines is inversely correlated with IQ, including up-regulation of IL-1β, IL-6, IL-10, and MCP-1 in autistic children with sleep disturbances and aggressive behavior [81,187]. A high MIF level combined with low CXCL10 was associated with executive functioning disturbance [187]. Adaptive and cognitive functions were associated with low TGF-β1 and GM-CSF without association with IL-10 or IL-12 [188,189,190]. A controversial but interesting difference between ASD subjects and healthy individuals included high peripheral brain derived neurotrophic factor (BDNF) level [136] and platelet-derived growth factor concentrations [188].

The transforming growth factors (TGFβ1, 2, and 3) play a core role in cell growth and differentiation, organ development, migration, matrix formation, and apoptosis, and are critical in the regulation of immune cells and cellular homeostasis [188,189,190]. In the context of the immune response, TGFβ1 during the early phase of development has the ability to enhance inflammation. TGFβ1 has been demonstrated to have a profound down-regulatory effect on T and B cell development and function, as well as the ability to modulate the differentiation and activation of NK cells, dendritic cells, monocytes/macrophages, granulocytes, and mast cells [134,176,190,191].

TGFβ1 derived from glial and neuronal cells and their receptors has a relevant distribution within the developing nervous system, giving to these cell populations a central role in the regulation of the normal brain development [192] and consequently in neurodevelopmental disorders due its involvement in neuroprotection via glutamate cytotoxicity, control of astrocyte differentiation and morphology, inhibition of astrocyte and microglia proliferation, cell migration in the cerebral cortex, control of neuronal death and microgliosis, and neuronal survival [134] (Figure 2). Finally, autistic children presenting dysregulated T-cell activities show an excessive production of cytokines (IL-5, IL-12, IL-13, IL-17, IL-21, IL-22, and IL-23) and a skewed CD4/CD8 ratio associated with decreased executive function, while subjects possessing Th1 endophenotype correlate with a more severe ASD behavioral phenotype [68,76,188] (Figure 2). Additionally, a reduced apoptotic signal (Fas) receptor CD95 together with increased HLA-DR and CD26 suggest a persistent peripheral T cell activation, and it has been found that circulating CD4+ CD25 high Tregs are functionally impaired, particularly in children with clinically severe form of ASD [191].

### 4.3. Immune Dysregulation and Comorbidities with High Incidence in ASD

Among the current findings in ASD, studies underline the coexistence of immune-mediated comorbidities that could predispose one to later-life autoimmunity linked to more aberrant behavioral conditions and the incidence of a major disease severity. Allergies, type I diabetes, GI dysfunction and inflammation, celiac disease, gut dysbiosis, obesity, seizures, and sleep disorders are the most frequent comorbidities contributing to aberrant behaviors in autism [193]. In this review, we will mainly discuss three of them. 

Allergic diseases are over-represented in ASD, and asthma is more common in children in early life who have an increased risk of developing ASD showing a major disease severity and deficits of socialization [194,195,196,197]. It has been also argued that food allergy may play a role in GI dysfunction in autism, and although it remains controversial, some parents of children with ASD have seen a behavioral improvement after implementing diets without gluten and casein [198]. There is a high incidence of non-IgE-mediated food allergy in younger children with ASD, and a correlation (although without supporting evidence) of GI symptoms and IgE-mediated food allergies has been reported [195,197]. The immunopathological mechanism underling this comorbidity in autistic children may be mediated by immunoglobulins and several neuropeptides derived from activated mast cells and basophils that release cytokines, which can in turn trigger enteric neurons as well by the mast cell–neuron interaction at the gut level. This could potentially influence behavior following the activation of enteric neurons through the vagal and spinal afferent pathways into the CNS [198]. Increased risk of allergies (allergic rhinitis, atopic dermatitis, and urticarial), type 1 diabetes, and Crohn’s disease have been also reported in association with ASD [192,199,200].

Regarding the molecular basis of comorbidities in infants with ASD, some authors have shown consistent evidence about the dysregulation of multiple innate signaling pathways overlapping comorbidities in ASD and coexisting with a high incidence of the disorder [201,202]. In this context, three innate immune pathways may be involved: the toll like receptor (TLR), nucleotide-binding, and oligomerization domain pathways, and chemokine signaling pathways. These pathways significantly overlap with both the pathology of asthma and inflammatory bowel disease [203,204].

Another one of the most commonly reported comorbidities in ASD is gastrointestinal (*GI*) tract pathology, which is present in approximately 50% of cases [205]. This intestinal dysfunction may be characterized by an increase in inflammation characterized by an epithelial infiltrate of monocytes, lymphocytes, NK cells, eosinophils, and intraepithelial lymphocytes, as well as the presence of IgG autoantibody and co-localized C1q complement binding in the basal membrane of GI epithelial cells [206,207,208]. Additionally, an increased mucosal production of inflammatory cytokines, including IL-17 and diminished regulatory IL-10 production, is marked as a factor contributing to the local dysregulation of mucosal immune responses and disruption of the intestinal barrier [209,210,211]. So, an upregulation of IL-17 cytokine production was observed in autism associated with an increased immune activation in ASD children with GI pathology [204,210]. A meta-analysis study found that the reported range of prevalence of these symptoms in individuals with ASD ranging between 9% and 91% [212]. In these studies, GI symptoms were abdominal pain or discomfort, constipation, diarrhea, and bloating [7,72,110], which showed a strong correlation with autism severity. Other studies looked toward the disturbed immune dysfunction and its connection with the gut microbiome and the brain [204]. 

A proposed mechanism of how the microbiota-gut-brain axis may have pathological involvement in neurological disorders considers that circulating antigens and bacterial metabolites directly influence the brain by disruption of both the intestinal and blood–brain barriers [213]. Evidence from individuals with ASD showing alterations in the blood–brain barrier and deficiencies in gene expression of intestinal tight junction proteins have been observed in autistic children, while other studies have also demonstrated dysbiotic alterations in gut flora and altered bacterial metabolites in these patients [43,209,210,213]. 

On the other hand, recent findings in children with ASD who exhibit GI symptoms were able to show marked differences in microbiota compared to children with a poorly regulated production of inflammatory cytokines and preferred bacterial growth [115], although authors were not able to give a consensus on whether the dysbios in ASD occurs as part of the immune dysfunction of autism itself or is a result of it. So, clinical manifestations such as diarrhea, constipation, and gastroesophageal reflux are major comorbidities in autism associated with mucosal inflammation, showing no favorable local innate immunity [68,72,110]. 

In addition, ingested allergens by the mother can elicit the secretion of several cytokines, such as IL-1β, IL-4, IL-5, IL-6, IL-12, IL-13, IFN-γ, and TNF-α, by mast cells and dendritic cells, which can reach the brain across the blood brain barrier (BBB) or stimulate the brain endothelial cells to induce a local immune response [194,195,196]. In our opinion, this confirms the innate immunity signaling as a main factor to dysregulate the brain environment leading to aberrant behavior in autism [115]. The gut microbiome can also directly contribute to sustaining inflammatory processes in the mother, to compromise, in turn, offspring development by their influence on microglia functional activity [214,215]. Gastrointestinal pathology in ASD has been also demonstrated by a higher number of helper and cytotoxic T lymphocyte infiltrates and CD19-positive B cells at duodenum, ileum, and colon in biopsies of children with ASD compared to histologically non-inflamed controls. This has been shown to be more enhanced at terminal ileum and colon level [199,206]. 

ASD frequently occurs together with epilepsy and sleep disorder, and the difference in cytokine profiles associated with epilepsy could be substantial, as was described previously by our group, which compared ASD children with and without epilepsy [83]. Electroencephalography (EEG) measures were used to classify individuals with or without epilepsy. Higher levels of IL-12 p40 were found in autistic patients with history of epilepsy, while IL-6 levels were higher in patients without epilepsy. This last piece of evidence was underscored by interictal epileptiform activity focused on the frontal brain region [83]; however, more studies are necessary in this area. Interesting data about sleep disturbances and aggressive behavior were found to be associated with up-regulation of IL-1β, IL-6, IL-10, and MCP-1 in autistic children [7]. Other studied areas with probably less incidence in autism are the fine motor skills (down-regulated IL-5, up-regulated MCP-1, RANTES, and eotaxin), visual reception (up-regulated IL-8, MCP-1, RANTES, and eotaxin) [138,205,216], and adaptive and cognitive functions (highMCP-1, RANTES, and eotaxin, low TGF-β1, and GM-CSF) [66,134].

## 5. Obesity, Fatty Acids, and Risk for Autism Pathology

During pregnancy, the immune system must ensure protection from external pathogens and prevent the rejection of the semi-allogenic fetus. During implantation, when the blastocyst adheres to the uterine epithelium and starts to invade the endometrium, an inflammatory reaction associated with an upregulation of IL-6 and TNF, among other soluble factors, allows for the repair of the uterine epithelium following blastocyst invasion [205,217]. After the parturition begins, an upregulated pro-inflammatory signaling involving IL-6 and TNF influences its progression [218].

Maternal obesity is associated with the development of a variety of neuropsychiatric disorders, although the mechanisms underlying this association are not fully understood. Maternal obesity has been revealed to be associated in a similar manner with the MIA maternal cytokine profile. However, how maternal cytokine levels, fatty acids, and placental inflammation might interact with fetal neurodevelopment to change microglial behavior and to cause epigenetic modification is left unanswered. Obesity is associated with chronic low-grade inflammation, which becomes increased during pregnancy with the risk for the development of neuropsychiatric disorders in offspring [219]. In this context, it is crucial to understand the mechanisms underlying the transgenerational effect of maternal obesity on brain development. This should be done without excluding the fact that during the highly dynamic neuroplasticity of prenatal fetal period, neural cells appear to be highly sensitive to both genetic and environmental stress [220,221]. 

Infection during pregnancy is associated with increased risk of neurodevelopmental and neuropsychiatric disorders, such as ASD; however, maternal obesity and mental disorders overlap with risks from infection to introduce a differential susceptibility of the brain to behavioral development and the consequent risks of developmental disorders respectively [222]. Other experimental evidence has been accumulated regarding the dysregulation of the immune system and obesity. Meta-inflammation, an inflammatory response underlying chronic and low-grade metabolic signals, may engage inflammatory pathways in adipose tissues, which may lead to increased immune cell infiltration [223]. In this case, although the placenta itself produces cytokines that might enter fetal circulation and impair brain development, it is not clear how maternal obesity affects this process [223]. In addition to these findings, associated genes could converge on hypoxic stress and immune pathways in the placenta to be key in ‘malprogramming’ the brain for later dysfunction. Studies of associations between cytokine genetic polymorphisms and neuropsychiatric disorders will be useful to help us better understand the real link between higher cytokine levels and risk of autism development. 

It is also known that various mechanisms that impact microglial biology during maternal obesity have shown that the adipose-derived hormone leptin regulates the microglial activation state via inflammatory cytokine production [224]. In this context, the increased levels of inflammatory cytokines found in an obese woman during pregnancy have been observed to be accompanied by an altered profile of circulating metabolites, including fatty acids [224,225]. Immunomodulatory properties of saturated and polyunsaturated fatty acids (SFAs/PUFAs) have been shown in autistic children, and relevant mechanisms of SFA-induced activation to resident macrophage populations in the CNS have been seen [226]. It is believed that SFAs cause macrophage activation as agonists of toll-like receptor 4, which induces changes in macrophage metabolism and induction of pro-inflammatory cytokine release from placental trophoblast cells. In both cases, these contribute to neuropathology in neurodevelopmental disorders [226,227].

Another relevant prenatal factor influencing the risk of autism pathology is related to maternal nutrition. Nutrition, essential to the maintenance of life, is key at the onset of life during the prenatal and early life periods of development of organs and systems and is crucial during pregnancy for the healthy development of the offspring [214]. Inadequate consumption of macronutrients by pregnant and/or lactating mothers leads to a diet with high levels of fats (regardless of their saturation state) and can induce maternal overweight or obesity, and diabetes, which coexist with a proinflammatory state characterized by increased release of IL-2, IL-4, and IL-6 cytokines [215,223,228]. Experimental evidence in animal models with a maternal high-fat diet has shown behavioral changes in the mothers with a negative impact on the offspring’s maternal care [223,229]. A reduced placental production of IGF-1 can also reduce the transfer of nutrients across the placenta to the fetus [165,166,167,168].

## 6. Immune Signaling Pathway Overlapping Autism and Neurodegenerative Disorders

Neuropsychiatric and neurodegenerative disorders display a biologically defined expression related to brain dysfunctions and age-related disease onset. The former, considered as a disturbed behavior and emotional state derived from the functional brain impairment, and the latter, viewed as an organic brain disease where the symptoms follow the damage of specific brain regions. Studies from different groups show biological evidence for the presence of common immune-mediated mechanisms overlapping both disease processes, although understandably with some distinctive characteristics.

Clinical and experimental evidence have argued similar mechanisms of innate immunity pathway signaling overlapping immune-pathological events in both neuropsychiatric and neurodegenerative disorders, characterized by the common influence of resident glial cells mediating inflammation via soluble molecules (mainly cytokines, chemokines, and complement proteins), which promote the recruitment of local immune cells and others coming from the peripheral compartment. To show this evidence, we refer to two pathologies occurring in the both extremes of the life: ASD, the main object of this review, and Parkinson disease (PD), following the main aspects of innate immunity relevant to both disorders and where the glial cells are the main cellular element.

In general, both disorders, ASD and PD, are related to brain dysfunctions, and in their particular context, genetic causes and risk factors play a central role in disease pathophysiology, severity, and disease progression besides the overlapping immunopathological mechanisms and molecular pathways. More than 100 candidate genes identified in ASD may converge as causal factors related to neuronal development, plasticity, synaptic structure, and performance [230,231]. Several genes and genomic regions, including alpha-synuclein (SNCA), parkinRBRE3 ubiquitin protein ligase (PARK2), chromosome 22q11deletion/DiGeorge region, and fragile X mental retardation 1 (FMR1) repeats, may be relevant to the development of both ASD and PD, with converging features related to synaptic function and neurogenesis. Both PD and ASD also show alterations and impairments at the synaptic level, representing early main disease phenotypes converging upon mechanisms active in the two diseases [232].

In ASD, three well-known core symptoms: impairments in social interaction; communication impairments; and restricted, repetitive, and stereotyped patterns of behaviors [233], can be accompanied by motor abnormalities, gastrointestinal problems, epilepsy, intellectual disability, or sleep disorders [205,216]. Similarly, in PD, the wide presence of non-motor symptoms, such as autonomic dysfunction, cognitive impairment, sleep disturbances, and neuropsychiatric symptoms, including depression, anxiety, and repetitive or obsessive-compulsive behaviors, are seen. These accompany the accumulation of intracellular protein reactive mainly for the alpha-synuclein (alpha-syn) and alpha-syn phosphorylated at position S129, and are a characteristic hallmark of the disease [217,218].

In PD, dopaminergic neurons project from the substantia nigra into the striatum [219], while in ASD the regions more affected include the frontotemporal and frontoparietal regions, the amygdala-hippocampal complex, cerebellum, basal ganglia, and anterior and posterior cingulate regions, and the most vulnerable neurons are those ubicated in the mentioned areas [220]. The loss of dopaminergic signaling in the nigro-striatal pathway has become the primary etiology for the motor disability in PD and the imbalance in the neuronal circuits of basal ganglia. These are also altered in ASD to affect both cognition and motor function [38,39]. In addition, impairment in cortical control of striatal circuits may be responsible for either the impulsive or compulsive symptoms observed in both PD and ASD [221,222], as well as for the clinical overlapping of cognitive and behavioral profiles, manifested by a high prevalence of depression and anxiety in both disorders, although this is more relevant to PD [223,234].

### Immunopathology in Both ASD and PD

It is emphasized that the microglial activation induced by a particular insult is conducive to neurotoxicity as a principal immunopathological mechanism taking place in both ASD and PD.

In psychiatric diseases, genetic susceptibility always fits with environmental factors, sometimes represented by intercurrent infections, toxic environmental substances, or other pathogens that stimulate the inflammatory response in a period in which the correct brain architecture is being built (childhood or even intrauterine life), giving rise to neurodevelopmental disorders. In this case, neurotoxicity follows from susceptible neurons being impacted by the glial inflammatory reaction, cytokines, and the oxidative stress reaction, which leads to damage and neuronal death, as has been written previously in this review.

PD experimental models and recombinant adeno-associated viral vector-based alpha-syn in rodent and primate models have revealed that abnormal deposition of misfolded alpha-syn can initiate neuroinflammation before the neurodegenerative process in PD [235,236]. Studies in humans have also demonstrated the presence of reactive microglia to HLA-DR class-II antigen in brain regions of PD associated with alpha-syn immunoreactivity [224]. Also seen is the increased expression of inflammatory mediators involved in microglial activation (CXC-family chemokine ligand 12, and its receptor CXC-family chemokine receptor 4, TNFα, IL-1β, IFN γ, nitric oxide synthase, and reactive oxygen species) in nigral tissue [225], as well as of proinflammatory interleukins (IL-1β, IL-2, and IL-6) and TNFα in striatum [226,227]. The presence of inflammatory mediators (IL-1β, IL-6, and TNFα) in cerebrospinal fluid and blood was also identified in PD [227]. Astrocytes have also become additional important glial cells linked to the microglia and give neurotrophic support for the control of synaptic homeostasis, which under pathological conditions may also show relevant proinflammatory and phagocytic activity, mediating neurotoxic damage and neurodegeneration in PD [214]. 

Some of the most important genes linked to familial forms of PD are highly expressed in microglia and astrocytes, which adds major susceptibility to PD pathology. The neurotoxic activity of A1-activated astrocytes has been identified in post-mortem tissue of PD patients, as evidence of their role promoting neurodegeneration in this disease [215,228]. Finally, is also well known that the phenotypic conversion of A2 protector astrocyte to A1 astrocytes with neurotoxic activity is actively promoted by reactive microglia through IL-1, TNF, and C1q factors [215] and is relevant to both ASD and PD. 

## 7. Conclusions

From this review, the current challenge in ASD lies in understanding the connection between immunological abnormalities, either as a risk factor for neurodevelopmental disorders or to devise a more effective strategy for management of the disease. It will lead to the elimination of some discrepancies seen in different studied populations, as well as provide a new clinical and experimental point of view, which will be useful to propose more attractive solutions to lead the way towards novel interventions.

## Data Availability

Not applicable.
